# Myasthenia Gravis: Novel Findings and Perspectives on Traditional to Regenerative Therapeutic Interventions

**DOI:** 10.14336/AD.2022.1215

**Published:** 2023-08-01

**Authors:** Evelyn Jou-Chen Huang, Meng-Huang Wu, Tsung-Jen Wang, Tsung-Jen Huang, Yan-Rong Li, Ching-Yu Lee

**Affiliations:** ^1^Department of Ophthalmology, Taipei Medical University Hospital, Taipei, Taiwan.; ^2^Department of Ophthalmology, School of Medicine, College of Medicine, Taipei Medical University, Taipei, Taiwan.; ^3^Department of Orthopedics, Taipei Medical University Hospital, Taipei, Taiwan.; ^4^Department of Orthopaedics, School of Medicine, College of Medicine, Taipei Medical University, Taipei, Taiwan.; ^5^Division of Endocrinology and Metabolism, Department of Internal Medicine, Linkou Chang Gung Memorial Hospital and College of Medicine, Chang Gung University, Taoyuan, Taiwan.; ^6^International PhD Program for Cell Therapy and Regeneration Medicine, College of Medicine, Taipei Medical University, Taipei 11031, Taiwan.

**Keywords:** Myasthenia gravis (MG), autoantibodies, acetylcholine receptor (AChR), Experimental autoimmune myasthenia gravis (EAMG), stem cell, exosomes, artificial intelligence (AI)

## Abstract

The prevalence of myasthenia gravis (MG), an autoimmune disorder, is increasing among all subsets of the population leading to an elevated economic and social burden. The pathogenesis of MG is characterized by the synthesis of autoantibodies against the acetylcholine receptor (AChR), low-density lipoprotein receptor-related protein 4 (LRP4), or muscle-specific kinase at the neuromuscular junction, thereby leading to muscular weakness and fatigue. Based on clinical and laboratory examinations, the research is focused on distinguishing MG from other autoimmune, genetic diseases of neuromuscular transmission. Technological advancements in machine learning, a subset of artificial intelligence (AI) have been assistive in accurate diagnosis and management. Besides, addressing the clinical needs of MG patients is critical to improving quality of life (QoL) and satisfaction. Lifestyle changes including physical exercise and traditional Chinese medicine/herbs have also been shown to exert an ameliorative impact on MG progression. To achieve enhanced therapeutic efficacy, cholinesterase inhibitors, immunosuppressive drugs, and steroids in addition to plasma exchange therapy are widely recommended. Under surgical intervention, thymectomy is the only feasible alternative to removing thymoma to overcome thymoma-associated MG. Although these conventional and current therapeutic approaches are effective, the associated adverse events and surgical complexity limit their wide application. Moreover, Restivo et al. also, to increase survival and QoL, further recent developments revealed that antibody, gene, and regenerative therapies (such as stem cells and exosomes) are currently being investigated as a safer and more efficacious alternative. Considering these above-mentioned points, we have comprehensively reviewed the recent advances in pathological etiologies of MG including COVID-19, and its therapeutic management.

## 1. Introduction

**Figure 1.**
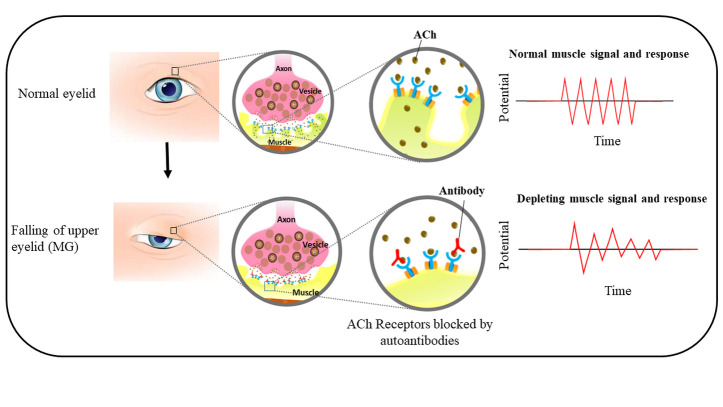
**The pathophysiology of MG, in which ACh Receptors are blocked by autoantibodies leading to depleted muscle signal and response**. MG: myasthenia gravis. Ach: Acetylcholine.

Myasthenia Gravis (MG) is an autoimmune systemic disorder in which autoantibodies are mainly produced against acetylcholine receptor antibodies (AChR), low-density lipoprotein receptor-related protein 4 (LRP4), or muscle-specific kinase autoantibodies (MuSK) leading to generalized or localized weakened muscular system including ocular, bulbar, and fatigue (Fig. 1) [1-3]. In addition, other clinical symptoms such as ptosis, diplopia, dysarthria, dysphagia, and proximal limb weakness may occur during a severe myasthenic crisis [4]. MG prevalence has increased throughout the world and its management is challenging to reduce the social and economic burden. The demographics also affect the risk of MG which is prevalent among many ethnic groups [5], particularly Africans. A retrospective clinical study reported that high jitter and decrement of various clinical symptoms/signs and disease severity imply severe MG conditions with bulbar and limb muscle weakness [6], and in the diagnosis of generalized or ocular MG the repetitive nerve stimulation has been equally effective [7].

Further, patients’ responses to the treatment of MG might vary according to population types. Management of MG is mostly based on corticosteroids, which are associated with adverse events and risks. Moreover, the co-morbidities as well as the recent outbreak of COVID-19 render MG management more difficult. The progress in understanding the mechanisms of MG and recent development in therapeutic approaches seem prospective to tackle the challenges. Immunosuppressive drugs are the second choice of treatment to stabilize MG conditions [8]. In life-threatening conditions, plasma exchange and intravenous antibodies are considered effective therapy for acute MG [9]. For MG patients with thymoma, the preferred therapeutic choice is thymectomy as it reduces the load of tumor cells [10]; however, it elevates the risk of the increased level of autoantibodies against AChR [11].

Recent development in targeted immunotherapy and stem cell therapy are considered potential approaches with improved efficacy, safety, and rapid response [12]. Most of the novel immunotherapeutic candidates target B-cells and complement systems involved in the generation of autoantibodies and autoimmune responses [13]. Attempts are also being made to understand the role of traditional Chinese medicines and herbs (TCM/TCH) along with the development of biomaterials such as platelet-derived biomaterials and others to improve therapeutic efficacy and safety. Considering the significance of these therapeutic approaches, this article provides a comprehensive insight into therapeutic progress for the management of MG.

## 2. MG: An Insight into epidemiology, etiology, pathophysiology, and diagnosis

### 2.1 Epidemiology

An increasing incidence of MGs has been documented in qualitative studies [14]. Approximately 700,000 individuals worldwide are affected by MG while 36,000-60,000 patients are estimated to be present in the United States of America (USA) alone [2]. The global incidence varies from 0.3 to 2.8 per 1,00,000; whereas, the median prevalence rate is 10 per 1,00,000 [15]. The reported global incidence rate of MG per 1,00,000 in various geographical regions are 0.4, 2.1, 1.9, 0.69-0.87, 0.69, and 2.1 for Norway, Italy, Australia, Japan, Korea, and Taiwan, respectively [15-21]. Reportedly, the incidence rates of acetylcholine receptor (AchR)-antibody-positive MG varies from 4.1 to 24 per million person-years in early and late onset, respectively throughout China; whereas, the incidence rate of AChR antibody-positive MG varies from 4 to 18 per million person-years [22-24]. A small population study reported MG incidence is 38.8 per million people in Argentina [25]. Similarly, the heterogeneous prevalence of MG ranging from 1.5 to 17.9 exists throughout the globe [14]. A cross-sectional study among Norwegians and the Dutch population indicates a higher incidence rate of AchR MG, muscle-specific kinase (MuSK MG), and ocular MG among the Norwegian population [26]. With increasing age, the incidence of MG is bimodally distributed among females, whereas linearly in males [14, 24, 27]. The prevalence and incidence rate of MG varies according to the subtype of MG and biological determinants such as age, gender, ethnicity, and difference in inherited genetics seem to contribute to this heterogeneous spread [14]. This has been implied in a recent study reporting a similar prevalence of MG among native European and Asian migrants. Remarkably, factors such as genetics, environmental factors, and lifestyles render Asian immigrants more prone to muscle-specific kinase antibodies MG and thymoma-associated MG (TAMG) [28-30]. In contrast, other evidence showed no significant difference in the overall incidence rate of seropositive MG among Southern African, European, and North American populations; which seems to overrule the role of environmental factors in the incidence pattern of MG [31]. Notably, the increased risk of MG among blood relatives of MG patients implies the inheritability of MG and its association with genetic factors [32]. It is evident that biological and environmental factors might be contributing to variations in the incidence and prevalence of MG, yet extensive scientific studies are required to clearly establish their role.

**Figure 2.**
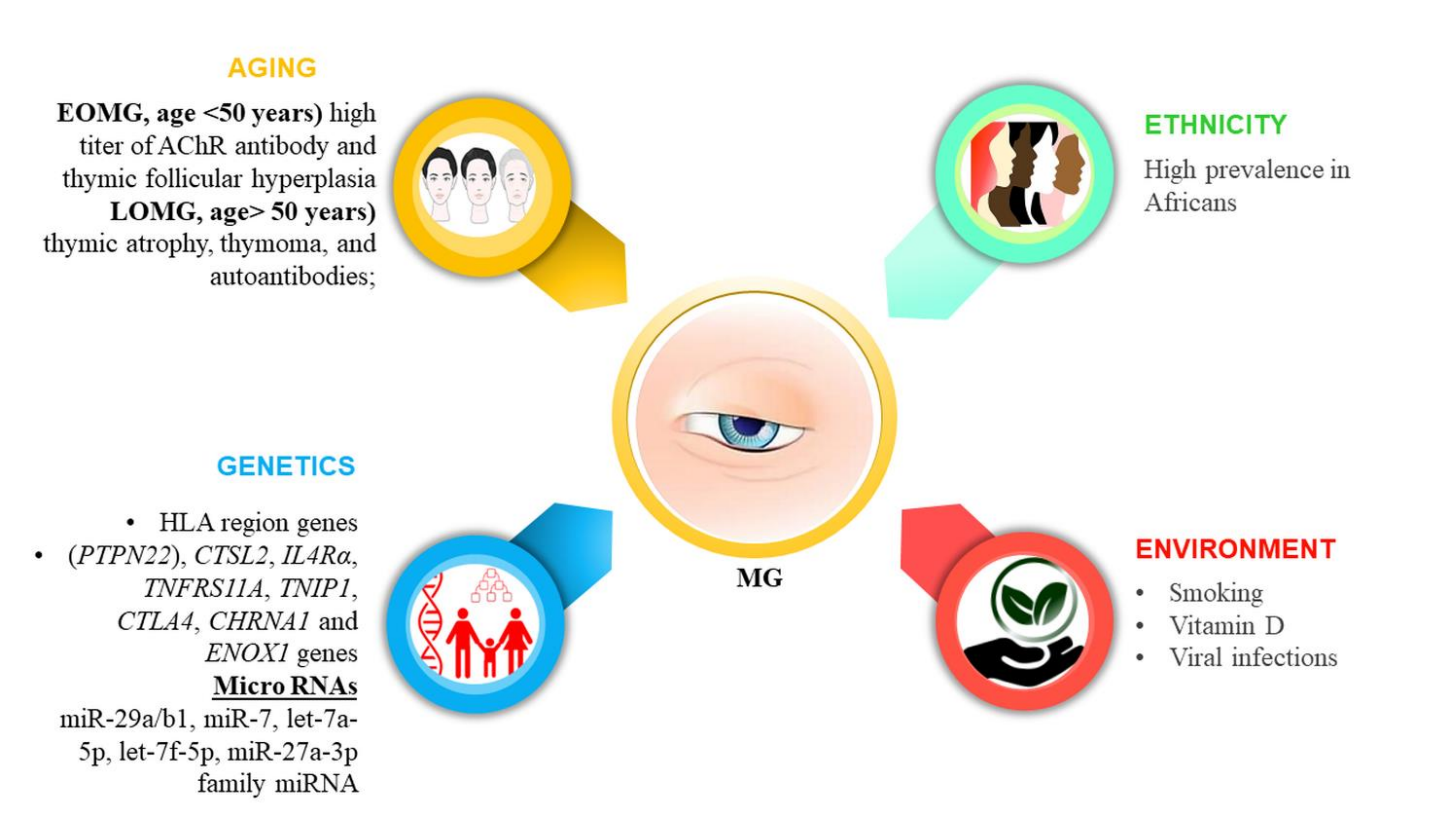
**Etiopathological factors of MG, which mainly include aging, genetics, ethnicity, and environment**. Based on the age, it is mainly of 2 types EOMG and LOMG. EOMG occurs at the age <50 years and is characterized by the high titer of AChR antibody and thymic follicular hyperplasia. On the other hand, the LOMG occurs at the age> 50 years) is distinguished by thymic atrophy, thymoma, and autoantibodies. EOMG: Early-onset MG, PTPN22: phosphatase non-receptor-22, *CTSL2:* cathepsin 2, *IL4Rα:* interleukin-4 (IL-4) receptor alpha, *TNFRS11A:* tumor necrosis factor superfamily IIA, *TNIP1:* TNFAIP3-Interacting Protein 1, *CTLA4:* Cytotoxic T-Lymphocyte-Associated Protein 4, and *ENOX1:* ecto-NADH oxidase 1 gene.

### 2.2 Etiological factors and pathophysiology

Understanding the etiological factors responsible for the initiation and progression of MG is crucial to establishing the pathological mechanism and developing effective and safe therapeutic strategies. MG is a multifactorial autoimmune disease, which is prominently impacted by etiological factors such as ethnicity, age, genetics (human leukocyte antigen (HLA), and environmental factors (Fig. 2). The HLA region genes are mostly present on chromosome 6p21, HLA A1-B8-DR3 haplotype, haplotype 8.1, HLA DQ alleles such as DQB1*0402, DQB1*0502, DQB1 *0503, and DQB1*0604 HLA DRB1*15:01 haplotype, HLA-DR14-DQ5 haplotype, HLA-DR14-DQ5, HLA-DR16-DQ5 haplotype, HLA-BS, HLA-A1, Dw3, DR3 and HLA-B12 [33-36]. The association of HLA haplotype with MG varies according to ethnicity, age, gender, and its subtypes such as early or late-onset MG [37]. Variations in protein tyrosine phosphatase non-receptor-22 (*PTPN22*), cathepsin 2 (*CTSL2*), interleukin-4 (IL-4) receptor alpha (*IL4Rα*), tumor necrosis factor superfamily IIA (*TNFRS11A*), TNFAIP3-Interacting Protein 1 (*TNIP1*), Cytotoxic T-Lymphocyte-Associated Protein 4 (*CTLA4*), *CHRNA1* and ecto-NADH oxidase 1 gene (*ENOX1*) genes are reported to be associated with MG [33, 38, 39]. Reportedly, the regulation of circulating micro-RNAs (miRNAs) such as miR-29a/b1, miR-7, let-7a-5p, let-7f-5p, miR-27a-3p family miRNA [40-44], and vitiating methylation of tumor suppressor genes have also been associated with MG and TAMG, respectively [45]. Apart from these etiologies, the additional factors including the overdose of drugs such as prednisone, D-penicillamine, anticholinesterase drugs, anesthesia, neuromuscular blockers for thymectomy, immune checkpoint inhibitors, interferons, tyrosine kinase inhibitors, cholesterol-lowering drugs such as statins, alemtuzumab, macrolides, fluoroquinolones, aminoglycosides, penicillins, blockers of β-adrenergic and calcium channel, antiarrhythmics, and magnesium has been found to induce MG [46, 47]. Vitamin D deficiency has been associated with MG progression [48]. A cross-sectional population study establishes that smoking was more prominent among MG patients in Norway [49]. Similarly, the severity of ocular MG was comparatively high among smokers than among non-smokers [50]. Additionally, another cross-sectional study among Norwegian pregnant women showed considerable association with postpartum duration [51]. Moreover, thymic inflammation has suggested the role of viral infection in the progression of MG [52]. Further, the Epstein-Barr (EBV) virus infection had been shown to present in MG thymuses [52, 53], and the prevalence of EBV-encoded small RNA-1 (EBER-1), Epstein-Barr virus nuclear antigen 1 (EBNA1), latent membrane protein-1 (LMP-1) in the thymus of MG patients support the role of EBV infection in MG progression. The EBV-induced TLR7/9 signaling pathway-mediated immune response might play a role in the progression of MG [54]. However, Meyer et al. and Jing et al. reported no evidence of EBV association with MG thymus contradicting the previous studies [55, 56]. Other studies also indicate the role of cytomegalovirus, human foamy virus (HFV), and Nile virus in the progression of MG [33, 57]. Paternal change in presence of antiviral biomolecule also indicates the incidence of viral-mediated MG.

MG is classified into early-onset, late-onset, ocular, and muscle-specific tyrosine kinase, LRP4 antibody-associated subtypes. Early-onset MG (EOMG, age <50 years) is characterized by the high titer of AChR antibody and thymic follicular hyperplasia which is more prevalent among women [9, 40, 58]. Late-onset MG (LOMG, age > 50 years) is distinguished by thymic atrophy, thymoma, and autoantibodies; whereas the ocular MG (OMG) is related to ocular muscles like extraocular muscles, levator palpebrae, and orbicularis oculi leading to the development of ptosis and diplopia [59, 60]. The symptoms represent a minimum of 2 years and nearly 15% of ganglionic AChR (G-AChR) are related to OMG [40, 61]. The MuSK MG is characterized by the presence of autoantibody against MuSK protein, which impacts bulbar muscles and occurs mostly in the early phase of life accounting for 5-8% of MG [9, 62]. LRP4 antibody-associated MG (LRP4-MG) is mainly characterized by the presence of antibodies against LRP4 [5]. Additionally, the presence of autoantibodies against molecules such as agrin, collagen Q, cortactin, ryanodine receptor, and Kv1.4 potassium channel among MG patients has been detected [63]. Nevertheless, in some cases, reports revealed no autoantibodies against AChR, and MuSK among MG patients.

In MG, autoantibodies against AChR, MuSK, and LRP4 disrupt the development of miniature end-plate potential (MEPP) and endplate potential (EPP) in the neuromuscular junction essential for the development of myofiber action potential required for effective muscle contraction [5, 64]. The reduction in EPP amplitude is associated with loss of postsynaptic sensitivity for the neurotransmitter Ach due to lower functional AChRs density [65]. In addition, autoantibodies against AChR and other postsynaptic NMJ biomolecules are the determinants for functional AChR and the safety factor of neuromuscular transmission [65].

The AchR autoantibodies belong to IgG1 and IgG3 subtypes which activate the classical complement system resulting in the recruitment of membrane attack complex leading to membrane damage [66-68]. This destruction of the membrane decreases MEPP and EPP resulting in the loss of the sodium-gated channel [66]. Moreover, these autoantibodies also bind to the AChR, interfering with the binding of ACh with its receptor (AChR) and lowering the action potential. Further, impaired postsynaptic differentiation has been demonstrated in animal models of MuSK MG [69-71]. MuSK autoantibodies mainly belong to the immunoglobulin (Ig)G4 subclass, which blocks the assembly of the agrin-LRP4-MuSK complex [72]. Interruption of MuSK kinase signaling leads to slow disassembly of the postsynaptic AChR clusters [71]. A resultant decline in miniature end-plate potential (mEPP) and EPP amplitude results in the failure of muscle action potential and leads to fatigue [66]. In AChR MG, the compensatory presynaptic upregulation of quantal release doesn’t take place in MuSK MG. In addition, the potential of IgG4 to exchange Fab-arm led to the development of monovalent IgG 4 antibodies resulting in rapid progression of severe MG among mice model [73]. This pathogenic role of Fab-arm exchange of IgG4 has also been observed among MG patients [74, 75].

The other autoantibody involved in the progression of MG is the LRP4 autoantibody, the LRP4 binds to agrin [76]. This interaction of trans-membrane protein and neural molecule activates the signal for AChR clustering [77]. The presence of LRP4 autoantibodies with either AChR or MuSK autoantibodies considerably increases the disease severity [78]. The LRP 4 autoantibodies are mainly of type IgG1 and IgG2. The presence of anti-LRP4 antibody among double seronegative (dSN) patients and its prevalence among ocular MG shows its significance in disease progression [79]. The secretion of autoantibodies in MG is a T-cell-dependent process, therefore the regulatory T cells (Treg) might have some association with MG [80, 81]. Treg cells regulate the peripheral self-tolerance and subdue Teff cells [82]. Treg dysfunction, down-regulation of cytotoxic T-lymphocyte-associated antigen 4 (CTLA-4), receptor activator of nuclear factor-kappa B ligand (RANKL), Forkhead box protein P3 (FoxP3), and IL-10 indicate the role of Treg among MG patients [82, 83]. The agrin autoantibodies are also detected among MG patients in presence of other antibodies such as AChR, LRP4, or MuSk [84, 85]. The presence of agrin and LRP4 autoantibodies disrupts agrin-mediated AChR clustering [86]. Along with the above-mentioned autoantibodies, the presence of autoantibodies against intracellular and extracellular self-antigens such as voltage-gated potassium channel Kv1.4 [87, 88], Collagen Q (Col Q) [89], Titin, RyR, rapsyn and cortactin have also been reported [76]. The impact of autoantibodies on the progression and severity of MG is well-established but the mechanism through which the T- cell tolerance barrier is passed to generate autoantibodies still needs to be explored. Thus, the understanding of the cellular mechanism for bypassing self-tolerance could provide an effective tool to diagnose and treat MG.

### 2.3 Artificial intelligence (AI) in the management of MG

Machine learning (ML), a subset of artificial intelligence (AI) technology is extensively studied to predict various pathologies and therefore contributes to the accuracy of disease diagnosis [90]. Methods such as support vector machines, random forests, and others have been employed to evaluate the role of ML in autoimmune disorders.

A study used 22 factors (age, gender, BMI, AChR antibody, years diagnosed with antibody, MuSK antibody, seronegative, thymectomy, sleep apnea, sleep apnea number, non-invasive ventilation (NIV) support, NIV number, MG-QOL15, ESS, ESS is greater than 10, Pittsburgh Sleep Quality Index (PSQI), PSQI >5, Fatigue Severity Scale (FSS), FSS >36, MG ADL, and MG ADL bulbar subset score) to determine the MG patient through logistic regression (LR), Gaussian discriminant analysis (GDA), convolutional neural network (CNN), and random forests (RF) [91]. Of them, the CNN model revealed the most fitted model for MG prediction. Additionally, 3D-DenseNet-DL-based deep learning multi-model using computed tomography (CT) images have been employed to predict MG among thymoma patients with a mean area under ROC curve, accuracy, sensitivity, and specificity of 0.730, 0.732, 0.700, and 0.690, respectively [92].

Case-based reasoning (CBR), rule-based reasoning (RBR), and ANN have also been used in an integrated computer system (ICS) to develop models, interpretations, and diagnoses of neuromuscular diseases including myasthenia gravis [93]. EMG parameters like amplitude, duration, phase, and physio-psycho parameters such as muscular, cognitive, and psychological have been used as cumulative confidence factors in hierarchal structure to determine neuromuscular diseases. Reports indicate that hospitalization stay time may be evaluated using ML methods such as stochastic gradient boosting (SGB), least absolute shrinkage and selection operator (Lasso), ridge regression (Ridge), eXtreme gradient boosting (XGboost), and gradient boosting with categorical features support (Catboost). The five factors including thymoma, age, corticosteroid use, intravenous immunoglobulins, and Myasthenia Gravis Foundation of America (MGFA) classification are the more prominent in determining hospital stay time [94]. Though, the application of high-dose corticosteroids should be considered carefully to optimize the hospitalization time as it may compromise the impact of immunotherapy in the adjuvant setting [95]. The two key clinical indicators- eyelid distance, and sclera distance have been used to construct a semantic segmentation model to predict ocular MG (OMG) [96]. This segmentation model attains a mean Intersection-Over-Union (mIoU) value of 86.05% which indicates its potential to lower diagnosis costs. A combinatorial approach using ML multinomial model and Monte Carlo randomization has been assistive in identifying SNP markers such as cerebellum 4 precursors (CBLN4), potassium channel KCNH5, adenylate cyclase 8 (ADCY8), GAB2, LINC00290, ACO1, HLA-G, SIX1, HS6ST1, GALNT10, ONECUT1, HLA-DRB6, and autism susceptibility candidate gene AUTS2 for neurogenerative disorders including MG [97]. The finding suggests that the model was effective in capturing neurological disorders with 83.6% accuracy and also implied a possible correlation between liver disease and neurological disorders. Moreover, a random forest model was developed using factors such as BMI, age at onset of MG, duration, and subtype of MG, Osserman’s grade, presence of bulbar, forced vital capacity (FVC), negative inspiratory force (NIF), presence of cardiac or respiratory comorbidities, history of infection and respiratory failure were effective in predicting and respiratory failure with 81.8% sensitivity and 89.3% specificity [98].

**Figure 3.**
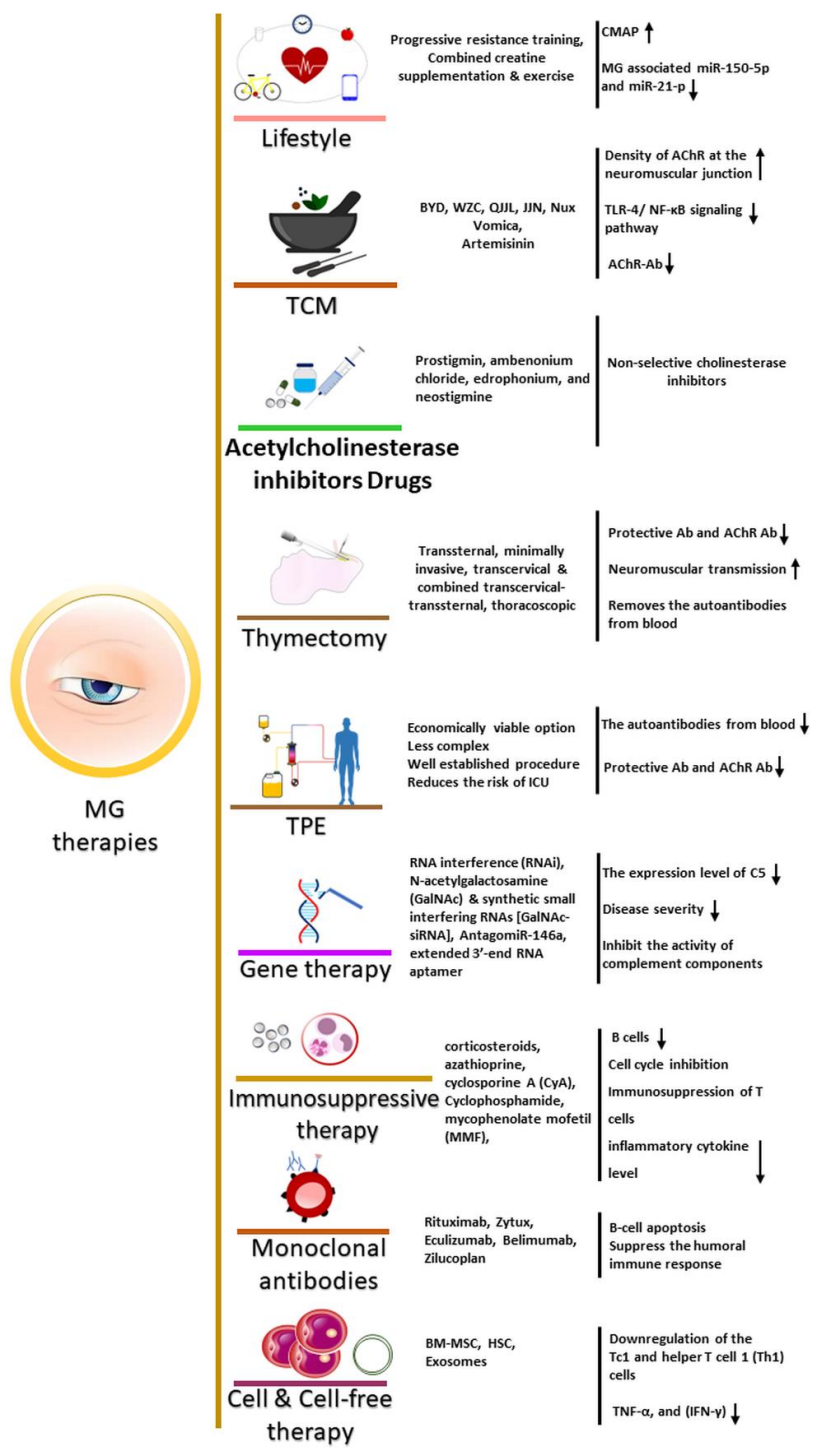
**Journey of therapeutic interventions for MG, which includes lifestyle factors, Traditional Chinese medicine/herbs, acetylecholinesterase drugs, thymectomy, TPE, gene therapy, immunosuppressive therapy, monoclonal antibodies, cell (stem cells) and cell-free (exosomes) therapy**. BYD: Buzhong Yiqi decoction, WZC: Wuzhi capsule, QJJL: Qiangji Jianli, grilled Nux Vomica, Jianpiyiqi, JJN: Jian Ji Ning, HSC: hematopoietic stem cell, (siRNAs) [GalNAc-siRNA]: N-acetylgalactosamine (GalNAc) and synthetic small interfering RNAs, TPE: Therapeutic plasma exchange, Tc1: Cytotoxic cell 1, Th1: Helper T-cell, Ach: Acetylcholine.

Collectively, the above studies show that AI and machine learning (ML) could be very helpful in not only predicting the MG but also managing it with improved efficacy and safety. Considering the significance of AI and ML, various studies have been conducted to develop protocols for the diagnosis and management of MG; however, their regulation and approval as the most effective method is still a challenge due to the lack of scientific and clinical data from different therapies and diagnosis methods.

## 3. Traditional, current, and emerging therapeutic approaches for MG management

During the last decades, the availability of more effective diagnostics, treatment, and intensive care has significantly improved the survival rate and lowered the mortality rate among MG patients (Fig. 3) [99]. Current approaches mainly include immunotherapeutic, immunomodulating, and immunosuppressive drugs such as corticosteroids, azathioprine, cyclosporine A (CyA), Cyclophosphamide, mycophenolate mofetil (MMF), tacrolimus (FK506) [100, 101]. These drugs are used to control autoimmune reactions. However, the associated risks and side effects in long-term use need to be considered carefully [102]. Along with immunotherapeutic drugs, other alternatives such as acetylcholinesterase inhibitors e.g., prostigmin, ambenonium chloride, edrophonium, and neostigmine promote the synthesis of acetylcholine [99, 101, 103]. The long-term therapy of MG with ACh inhibitors leads to significant adverse effects, [104], and notably only AChR MG patients properly respond, whereas other autoantibodies mediated MG such as MuSK MG patients don’t respond. Plasma exchange (PE) and intravenous immunoglobins (IVIG) are other available therapies used to provide rapid relief. Though the choice of treatment depends on cost, facility, complications, and comorbidities [105], thymectomy is considered as best resort to provide stable remission and the development in operative techniques has improved surgical efficacy and outcomes [106].

The recent developments in molecular and regenerative medicine have provided the option to explore a more effective and safer way to provide next-generation treatment. Considering the limitations of current therapies, attempts are being made to explore the potential of complement inhibition therapy [107], biomolecules targeting B-cell, T-cell, cytokine, and Fc receptor, novel antibody molecules, cell therapy including such as Chimeric Antigen Receptor-T (Car-T) Cell Therapy (CART), and nucleic acid technology like antisense technology to provide desired improved treatment for MG [12, 108]. In addition, the prospects of traditional medicine and regular exercise are also under evaluation for their role in improving the quality of life (QoL) of MG patients [109, 110]. These developments in current and novel treatments (Table 1) have been discussed in the next sections.

### 3.1 Physical Exercise and activities in MG treatment

Physical exercise is an effective way to live a healthy and active life in the normal population. It could also mitigate chronic autoimmune diseases like chronic obstructive pulmonary disease (COPD), inflammatory bowel diseases (IBD), systemic lupus erythematosus (SLE), rheumatoid arthritis (RA), and multiple sclerosis (MS) [111, 112]. A case study implied that exercise improves muscle strength, and endurance [113], muscle resistance, physical performance, and compound muscle action potential (CMAP) amplitude for biceps brachii and rectus femoris muscle in an MG patient [114]. Additionally, a reduction in the level of MG associated-microRNAs (miR-150-5p and miR-21-p) was recorded. Similarly, the physical exercise-induced improvement in CMAP, isometric muscle force, and muscle thickness of rectus femoris muscle among MG patients [115]. Although, no functional enhancement was observed in the biceps brachii muscle. A comparative study showed that progressive resistance training is more effective in enhancing muscular strength and function than aerobic training; however, with similar adverse events [116]. Interestingly, creatine supplementation with resistance exercise could promote an increase in fat-free mass and muscle strength in the MG patient [117]. Extensive exercise reduces muscle mass but ameliorates vital capacity, walking ability, and severity of symptoms [118]. Respiratory muscle training (RMT) has significantly improved (p<0.05) forced vital capacity (FVC) and forced expiratory volume in one second (FEV1) with a considerable reduction in the fatigue score (MFSI-SF) 17.1±14.7 to 13.5 ± 16.9 (p=0.03) in a single-center hospital-based prospective study [119]. Similarly, long-term respiratory muscle endurance training (RMET) significantly amelioration (p<0.001) in physical fitness, respiratory system, and MG symptoms by 64%, 58%, and 49% [120]. Continuous physical training of a minimum of 150 minutes per week is required for mild and moderate MG [110]. Overall, balance, physical, and respiratory training have been found effective in improving functional outcomes, QoL, and reduction in fatigue [121]. Thus, many studies have reported the efficacy and safety of physical exercise in the improvement of the quality of life (QoL) of MG. Nevertheless, it is not feasible to recommend due to the lack of high-quality clinical evidence and inconsistency in methodological approaches.

**Table 1:** Summary of various therapeutic approaches for MG.

Therapeutic approaches	Advantages	Limitations
**Physical Exercise and activities**	• Improves muscle strength and endurance. • Reduction in MG associated miRNA. • Improvement in CMAP	• lack of high-quality clinical evidence and inconsistency in methodological approaches
**Traditional Chinese medicine (TCM)/Traditional Chinese Herbs (TCH)**	• Improvement in the density of AChR *in vivo*. • Used as an immunosuppressant for the MG	• Lack of preclinical, clinical, and scientific studies. • Inconsistent formulation
**Cholinesterase Inhibitors**	• Provide symptomatic relief • Non-selective cholinesterase inhibitors	• Adverse events such as bradycardia, rash, hypersalivation, headache, blurred vision, tingling sensation in fingers and toes, and colicky pain • Need extensive clinical studies
**Immunosuppressants and steroid drugs**	• Immunosuppressive • Effective and found to be safe up to a certain limit	• Adverse events in long-term
**Thymectomy**	• Reduces the use of immunosuppressants and risk of hospitalization	• Need expertise and careful observation needed before recommendation
**TPE**	• Ameliorates neuromuscular transmission resulting in improved motor and muscle function	• A higher volume of plasma to process.
**Antibody-mediated Therapy**	• Effective in improving quality of life in severe as well as refractory MG	• Insufficient pharmacokinetics, impaired interactions with immune cells, and tissue accessibility
**IA therapy**	• Selectively screens pathogenic molecules, antibodies, and other undesired components of plasma.	The removal of fibrinogen, coagulating factor, and lack of sufficient clinical studies
**Gene therapy**	• Potential to inhibit the activity of complement components	Need more clinical and scientific studies for effective evaluation therapy
**Stem cells**	• Regenerative approach • Immunosuppressive potential	• Still in developmental phase and need more studies
**Exosomes**	• Biocompatible, lower antigenicity, and immunogenicity, regulation signalling pathways	• Need more clinical studies

MG: Myathenia gravis, CMAP: Compound muscle action potential, TPE: Therapeutic plasma exchange, IA: Immunoadsorption.

### 3.2 Traditional Chinese medicine (TCM)/Traditional Chinese Herbs (TCH)

TCM/TCH recognizes multiple etiological factors in the pathogenesis of MG, and accordingly, various therapeutic approaches are being recommended [122]. Of them, acupuncture (AcP) is an essential part of TCM which has been used either alone or in combination with other therapeutic alternatives to treat even MG [123]. In experimental models, AcP reduced the serum levels of tumor necrosis factor-α (TNF-α), interleukin-12 (IL-12), and interleukin-18 (IL-18) along with an increase in transforming growth factor β (TGF-β). [124, 125]. The AcP also improved the density of AChR at the neuromuscular junction in the rat model [126]. The active ingredients of TCH are rich in flavonoid molecules which could mimic the act of acetylcholinesterase inhibitors and provide therapeutic relief [127]. In addition, being economical, effective with enormous availability, and having low side effects render TCM/TCH a preferable candidate for MG [128]. Interestingly, a meta-analysis reported no significant difference in the efficacy of western therapy and Chinese medicine; however, their combination was found more effective than individual treatments [129]. One of the main objectives of TCM therapy is to re-strengthen the spleen and kidney, as any renal deficiency may exacerbate the deficiency of the spleen, which further worsens the symptoms of MG. [130]. Among the TCMs, the Buzhong Yiqi decoction (BYD) is considered an effective Qi-supplementing strategy in MG management., as this disorder is been associated with a Qi-deficiency pattern [131]. Further, a few systematic reviews and meta-analyses have indicated the clinical potential of BYD and its combination with western medicine [131, 132]. Another TCM known as Wuzhi capsule (WZC) has can enhance the blood concentration of Tacrolimus an immunosuppressant used for the MG [133]. The WZC contained components such as schisandrin A, schisandrol A, and schisandrol B of competitively inhibited the binding of Tacrolimus to the CYP3A4 and CYP3A5, thus reducing the metabolism of the drug. Artemisinin, a TCM for Malaria has also been found effective in EAMG by significantly reducing the level of TNF-α and IL-17^+^ cells, along with an increase in TGF-β1 and regulatory T cells (Treg) cells. Similarly, the artesunate, a semi-synthetic derivative of Artemisinin has been reported to exert immunomodulatory and anti-inflammatory in EAMG [134]. Similarly, Qiangji Jianli (QJJL) decoction may improve energy metabolism and regulate mitochondrial enzymatic activity [135]. This decoction modulates mitochondrial fusion and fission via activating AMP-activated kinase/ peroxisome-proliferator-activated receptor γ coactivator 1α (AMPK/PGC-1 α)[136]. Similar to these TCMs, the grilled Nux Vomica (seed of *Strychnos nux vomica L*,) improves EAMG characteristics in Lewis rats by downregulating the TLR-4/ NF-κB signaling pathway [137]. Granule of another TCM Jianpiyiqi may also suppress AChR-Ab and modulate cytokine and immune parameters leading to improvement in MG conditions [138]. Further, for a long time Jian Ji Ning (JJN), another TCM/TCH has been used for the management of MG. This is possible through regulating serum microRNAs (miRNAs) and inhibiting the apoptotic pathways of a few immune cells resulting in improved Quantitative MG Score (QMG)[139]. Taken together, TCM/TCH offers a significant therapeutic impact in improving MG conditions. Nonetheless, the lack of extensive preclinical, and clinical studies along with inconsistency in TCM/TCH formulation limits the wide acceptability of TCM/TCH.

### 3.3 Cholinesterase Inhibitors: A primary candidate for symptomatic treatments of MG

Prostigmin, (cholinesterase inhibitor) is one of the oldest drugs which provide symptomatic relief [99, 140]. Still, the drug has been replaced by pyridostigmine in the 60s, owing to its safer profile in the short and long term among non-progressive mild MG [141]. Notwithstanding, the non-selective cholinesterase inhibitors are preferred drugs and are mostly found effective among AChR-MG patients [142]. Though cholinesterase inhibitors are widely accepted first-line drugs, yet lack of randomized clinical trials indicates the need to conduct extensive studies to evaluate efficacy and safety incoherence among observational studies, case reports, and other clinical studies [143]. The common adverse events related to cholinesterase inhibitors are bradycardia, rash, hypersalivation, headache, blurred vision, tingling sensation in fingers and toes, and colicky pain. In a preclinical study, it has been demonstrated that C547 a chemically synthesized compound through selective inhibition of acetylcholinesterase reduces the risk of hyperactivity in the urinary bladder [144]. C547 is a slow-binding inhibitor of AChE and a prominent candidate for selective enzyme inhibition thereby improving the efficacy and safety of cholinesterase inhibitor drugs [145]. Additionally, quaternary ammonium salts, another selective inhibitor are being investigated in the pre-clinical studies to assess their pharmacological significance in MG [146]. Based on this evidence, the potential application of selective AChE inhibitors is considered the first-line defense for MG. More pre-clinical and clinical studies are required to demonstrate the effectiveness and safety of cholinesterase inhibitors.

### 3.4 Immunosuppressants and steroid drugs

Immunosuppressant drugs are the only choice for providing long-term therapy for MG as well as other autoimmune neurological disorders. The immunosuppressant drugs for MG are classified into three groups based on their function (i) Targeting and depletion of B cells (rituximab) (ii) cell cycle inhibition (azathioprine, methotrexate, mycophenolate mofetil, and cyclophosphamide) (iii) Immunosuppression of T cells (ciclosporin, tacrolimus, and steroids) [147]. The choice of therapies is challenging due to heterogeneity in pathogenesis, drug/dose response, and severity [148]. Long-term therapy of MG is mainly initiated with the combination of Azathioprine with steroids at the minimum possible dose [147]. Another immuno-suppressant like mycophenolate mofetil, tacrolimus, or methotrexate is being considered MG-resistant to Azathioprine treatment. In addition, the application of cyclophosphamide and Ciclosporin are carried out at the severe stage of MG, yet the use of drugs is associated with a higher rate of serious adverse events. Use of the early prednisolone (corticosteroid) lowers the risk of progression of ocular myasthenia gravis (OMG) to generalized MG [149]. The prednisolone dose is usually started with a dose of 1 mg/kg/day and subsequently, the dose is reduced to the lowest level [148]. The therapeutic effect of corticosteroid drugs is associated with the down-regulation of endothelial adhesion to leukocytes and lowering of the inflammatory cytokine level resulting in an inhibitory impact on immune response [99]. Further, the therapeutic efficacy of thymectomy and prednisone combination therapy was found higher than that of prednisone alone [150]. Out of various steroid immunosuppressant drugs, the standalone use of ciclosporin or cyclophosphamide in combination with corticosteroid has been significantly effective in MG [151]. Treatment with a considerably low dose of intravenous immunoglobulin reduces the need for a time-weighted average dose of prednisone drug [152]. Additionally, it has been suggested that the combination of low-dose prednisone with other therapeutic options in early treatment is effective in the remission of MG [153]. However, the current dose regimen is not equally effective in all MG patients. Steroids/corticosteroids are found to be effective, yet the lack of randomized clinical trials (RCTs) and the adverse effects of steroids needs to be addressed for their wide and safe application. Notably, Azathioprine reduces the dose of steroids and is mostly recommended at a dose of 2-3 mg/kg/day. A combination of changes in prednisone dose and Azathioprine has been shown tolerable and affordable with improved outcomes among severe and moderate MG [154].

Azathioprine is metabolized into 6-thioinosinic acid and 6-mercaptopurine, which intervene in T cell function by suppressing purine synthesis [148]. In addition, thiopurine S-methyltransferase (TPMT) genotype polymorphism has been closely associated with azathioprine-related adverse events. This genetic variation is the only significant factor for serious adverse events and not for minor [155]. Notably, the risk of cancer is increased among MG patients, during prolonged use of azathioprine [156]. Cyclophosphamide is another immunosuppressive agent which has been used in the case of MG remission. In a six-month follow-up, cyclophosphamide has been found effective in improving symptoms of refractory MG [157], and when applied in high doses, cyclophosphamide could restore normal activity in severe conditions of refractory MG [158]. The major adverse events such as the high risk of infection, severe toxicity, malignancy, and bone marrow suppression are associated with the long-term use of cyclophosphamide, therefore need to be carefully evaluated before recommending the drug [147, 159]. Moreover, cyclophosphamide is also recommended after the application of other immunosuppressant drugs in severe refractory MG. Conclusively, the immuno-suppressant and steroids are first-line treatment resorts and have been used for a long time. These drugs are effective and found to be safe up to a certain limit but the development of refractory MG and associated adverse events in long-term use need to be carefully considered before recommending them.

### 3.5 Thymectomy

The thymus is the source of plasma cells secreting AChR autoantibodies [160]. Surgical excision of the thymus is one of the common approaches to provide long-term therapeutic relief [106]. The presence of thymoma in MG patients strongly indicates a strong need for thymectomy to effectively control the further increase in titer of AChR antibodies. It also reduces the need for immuno-suppression and cases of hospitalization of exacerbations [161]. Transsternal, minimally invasive, transcervical, and combined transcervical-transsternal thymectomy are the major four surgical thymectomy techniques. A study reported thoracoscopic thymectomy as safer with improved neurological outcomes compared to the transsternal approach [162]. Though the presence of circulatory peripheral blood B-cells was detected even after 12 months post-thymectomy [160, 163], these cells pose the risk of MG remission. The safety and efficacy of thymectomy are affected by factors such as age, preoperative conditions, AChR Ab titer, and pathological conditions [163]. In patients aged above or equal to 42 years, the resection level and Masaoka-Koga stage > I increase the risk of relapse and poor clinical outcome of thymectomy [164]. In addition, Osserman stage (IIA-IV) and WHO type B2/B3 thymoma are independent risk factors for post-operative myasthenia crisis (POMC) [165]. Similarly, thymoma has been associated with the below expectation of stable remission [166]. In the recent decade, surgical techniques for thymectomy have been improvised through video and robotic assistance [167]. The video assistance thoracoscopic surgery (VATS) was much more effective in reducing hospitalization time, operative time, blood loss, and postoperative pain with improved remission as compared to transsternal surgery [168]. In addition, VATS also resulted in decreased demand for prednisone post-treatment [168]. Refractory MG is commonly associated with the presence of MuSK autoantibodies, history of thymoma/ thymectomy, and female gender [169]. Repeated thymectomy is safe, especially among patients who had undergone transcervical earlier, and improved the condition of refractory [170]. Left-sided thymectomy provides shorter operating times and improved outcomes compared to right-sided thymectomy [171]. The use of ultrasonic energy is now considered an alternative to bleeding during thymectomy [172]. Additionally, thymectomy has been found curative for aplastic anemia along with MG [173]. Further, the combination of prednisone and thymectomy provide comparatively improved relief from prednisone alone treatment [174]. Similarly, the outcomes of thymectomy in non-thymomatous ocular MG among children were better than in adults with complete stable remission [175]. Overweight and obesity increase the risk of longer hospitalization, postoperative respiratory failure, and other associated adverse events [176]. Respiratory muscle paralysis causes life-threatening event- like postoperative myasthenia crisis (POMC) and required ventilation [177]. In addition, exercise is not considered a contraindication of MG and rehabilitation can easily adopt post-surgery [178]. The presence of AChR Ab in a non-MG patient after thymectomy has been associated with the development of post-thymectomy MG [179]. Thymectomy is considered to provide long-term therapeutic relief in MG patients. The recent development in surgical techniques has improved outcomes of the surgical techniques. The surgical techniques have reduced the requirement for other drugs and found to enhance the efficacy and safety of this intervention. However, this option needs to be carefully evaluated before recommending the patients.

### 3.6 Therapeutic plasma exchange (TPE) therapy in MG treatment

TPE is a well-practiced procedure to suppress the disease progression, and weakness, as well as to prepare the patient for surgery and corticosteroids [180]. The TPE removes the autoantibodies from blood [181] and ameliorates neuromuscular transmission resulting in improved motor and muscle functions [182]. TPE reduces both the level of protective Ab and AChR Ab; additionally, it delays the need for immediate immunosuppressive therapy [180]. TPE seems an economically viable option for developing countries owing to its low cost and complexity [183, 184]. This therapy has been found effective for MuSK MG patients too [185]. Even a short-term TPE reduces the risk of ICU [186]; whereas regular TPE therapy is effective in improving quality of life and reducing the frequent need for immunosuppressants [187]. A clinical trial with an 8-year follow-up reported a safer and more efficacious profile of TPE sessions every 2-3 weeks for severe and moderate MG patients who were not unresponsive to immunosuppressive therapy [188]. Further, a comparative study found that a combination of TPE with immunoadsorption (IA) or IA alone reduced hospital stay with an improvement in MG conditions [189]. Further, TPE and intravenous (i.v.) immunoglobulin (Ig) therapy showed similar efficacy with comparable tolerability [190]; but with rapid recovery in the TPE group [191]. In a systemic review and meta-analysis, TPE was more effective than Ig (i.v.) among acute MG patients and patients undergoing thymectomy [192]. Moreover, the high frequency of TPE, dysphagia and glomerular filtration rate (GFR) was found to be associated with MG relapses, and low GFR [193]. Thus, the TPE is a reliable treatment approach for MG yet more studies are required to improve its clinical efficacy and safety along with improving the demands of therapeutic procedures. Consequently, the clinical effect of double filtration (DF) and IA was similar. However, IA was much more effective in clearing out AChR antibodies [194]. The tolerance level of PE and IA were almost similar. Contrary to the previous studies, a higher adverse event (AE) for IA has been reported [195]. The therapeutic effect of IA is mainly associated with an intravascular decrease in autoantibodies, immunomodulatory activity, and redistribution of antibodies. Whereas the higher incidence of AE for IA treatment has been associated with the need for a higher volume of plasma to process.

### 3.7 Antibody-mediated Therapy

Rituximab is a chimeric anti-CD20 monoclonal antibody (mAb) that mainly targets B cells by activating the complement system, thereby inducing cell apoptosis and antibody-mediated cytotoxicity to suppress the humoral immune response to provide therapeutic relief in MG [196]. The therapeutic efficacy of Rituximab has been well recorded against MuSK-Ab MG [197]. Recent clinical trials also indicate the efficacy of Rituximab in providing therapeutic relief in AChR-Ab MG and related myasthenic crises [198]; however, its therapeutic response against AChR-Ab MG is still open for discussion. Similarly, the efficacy of Rituximab has been found effective in both MuSK Ab and AChR Ab MG and is a considerable option for reducing prednisone drug load, and providing symptom-free clinical relief [199]. This treatment provides an additional advantage of rapid remission, low exacerbations, and decreased chance of hospitalization after treatment for MuSK MG patients. Notwithstanding, the effective use of Rituximab in MuSK-Ab MG is still off-label as it provides various advantages over traditional immunosuppressive drugs [200]. Treatment with Rituximab at an early stage of non-MuSK general MG has improved outcomes with better tolerability as compared to late treatment [154]. In a retrospective longitudinal study on refractory MG, Rituximab lowered the prednisone dose requirement and improved the clinical outcomes [201]. It is also efficacious in reducing the prednisone dose requirement in refractory MG [202]. In the long-term treatment with Rituximab, the level of specific IgG4 MuSK Ab could be reduced at a significant level yet the complete removal of IgG4 has not been detected even after 10 years of treatment [196]. The presence of a small level of IgG4 might be associated with plasma cells. Thus, recent studies indicate that Rituximab is effective in improving the quality of life in severe as well as refractory MG [203]. In addition, Zytux, a biosimilar to Rituximab is also effective in providing therapeutic relief for refractory MG [203]. Another mAb known as Eculizumab can mitigate the autoantibody effect on C5 protecting neuromuscular junction [204], and therefore is now being considered a safer therapeutic candidate. Further, other mAb i.e., Belimumab targets B lymphocyte stimulator (BLyS)/ B cell-activating factor (BAFF), used in the treatment of Systemic lupus erythematosus (SLE) is not much effective in improving Quantitative Myasthenia Gravis (QMG) score [205]. Additionally, peptides such as zilucoplan could be an effective tool in the treatment of MG due to their inhibitory ability of membrane attack complex through targeting the C5 component of the complement system [206]. Antibody therapy has come up as an effective tool to control the progression of MG but the functional limitations such as insufficient pharmacokinetics, impaired interactions with immune cells, and tissue accessibility need to be addressed [207].

### 3.8 Immunoadsorption (IA) therapy in MG

TPE and immunoadsorption (IA) have been utilized for the treatment of autoimmune neurological disorders like chronic inflammatory demyelinating polyneuropathy, Guillain-Barré syndrome, neuromyelitis optica spectrum disorders, autoimmune encephalitis, and MG [208, 209]. Still, IA is seen as an alternative to TPE due to being comparatively economical with a low risk of viral infection and anaphylactic reaction [210]. IA selectively screens pathogenic molecules, antibodies, and other undesired components of plasma. The traditional method of IA utilizes an affinity column with tryptophan or phenylalanine [210]. However, nowadays column of Sepharose B with protein-A or anti-human Ig sheep polyclonal antibody has been found as a suitable alternative to remove antibodies, which are mostly IgG [210, 211]. In TPE, the separated plasma is replaced with frozen plasma or electrolyte solution; while in IA, autoantibodies removed plasma is reintroduced in the patient without any additional requirements of frozen plasma or any other solution [211]. The protein A adsorbent column can remove nearly 60% of IgG and around 70% of autoantibodies against plasma [212]. It also removes the vitamin K-dependent coagulation factor though the coagulation value returned to normal after 24 hours. The monitoring of anti-Xa activity and coagulation factors is required before recommending low molecular weight heparin (LMWH) [213]. IA through a protein A column not only removes approximately 90% IgG but also autoantibodies against double strand (ds) DNA, glomerular basement membrane (GBM), cardiolipin, human leukocyte antigen (HLA), and circulating immune complex (CIC). The affinity of protein A column has been much higher for CIC as compared to IgG [214]. IA method was also effective in regulating humoral, cellular immune response, and T_H_ /Tc ratio. In a prospective randomized control pilot trial (PRCPT), both PE and IA reduce the level of AChR autoantibodies at a significant level even after 28 days but an increase toward the base level has also been recorded post-treatment [215]. During treatment, patients were also administered immuno-suppressive drugs. Conclusively, the use of both treatments 3 to 6 times has been reported as safe to reduce the life-threatening burden of MG crisis. Notably, IA has been recorded as comparatively safer than PE with a low number of serious and non-serious adverse events, and effective to provide long-term remission among MG patients with thymoma or thymic hyperplasia [216]. Further, long-term IA may reduce the dose of oral immunosuppressive drugs among MG patients resistant or contraindicative to drug therapy [217]. The specificity of IA has been optimized in a MuSK- experimental autoimmune myasthenia (EAMG) and found effective in removing MuSK antibodies from plasma [218]. Likewise, various pre-clinical and clinical studies have demonstrated the selectivity, efficacy, and safety of IA treatment; however, the removal of fibrinogen, coagulating factor, and lack of sufficient clinical studies are limiting factors for its wide acceptability.

### 3.9 Progress in Gene therapy

Complement (C)-mediated immune response and accumulation of membrane attack complex (MAC) have been detected at motor end plates [219, 220]. Therefore, targeting the complement system for therapeutic purposes may be considered [221]. In particular, RNA interference (RNAi) has been studied for its potential to inhibit the activity of complement components, which might be extended for their evaluation in EAMG. In an important study, a conjugate of N-acetylgalactosamine (GalNAc) and synthetic small interfering RNAs (siRNAs) [GalNAc-siRNA] was used to silence the expression of complement C5 in hepatocytes [221]. Repeated doses of GalNAc-siRNA resulted in a significant decrease in the expression level of C5 and disease severity. Moreover, this result has been confirmed in the resistance of MG induction among passive transfer MG (PTMG) models. the clinical safety and translation of GalNAc-siRNA silencing indicate *in vivo* potential for MG [222]. Further improvement in the design of the GalNAc-siRNA complex resulted in improved stability and efficacy [223]. Similarly, downregulating the expression of the C2 component of the classical pathway using C2-siRNA resulted in an improvement in the progression of EAMG [224]. The silencing of the RelB gene through Rel-B specific short hairpin RNAs (ShRNAs) in bone marrow-derived dendritic cells (BMDCs) reduced the frequency of EAMG and improved the circulation of T regulatory cells [225, 226]. In addition, B cell lymphoma 6 (Bcl-6) a transcription factor of follicular helper T (Tfh) cell has been silenced using siRNA-Bcl6. This gene silencing approach revealed a reduced level of Tfh cells, Bcl-6, IL-21, and anti-AchR antibody, and an improved severity of MG [227]. Silencing of miR-146a, a regulatory nucleic acid molecule, through AntagomiR-146a (single-stranded RNA inhibitor) may also relieve EAMG characteristics and divergence in B-cells [228]. Even the RNA aptamer with an extended 3’-end has been used as a decoy to trap mAb198 which binds to AChR and lowers the binding frequency of mAb198 to AchR present in human cells [229].

Further, antisense oligodeoxynucleotides of AChE mRNA form an RNA duplex with AChE mRNA, which results in RNases-mediated degradation of this RNA duplex with a consequent decrease of active AChE level [230]. Besides, the EN 101 antisense oligodeoxy-nucleotides were found to be effective in providing therapeutic relief and improvement in muscle strength, and clinical outcome [231-233]. Thus, these preclinical studies indicate the potential of RNA interference techniques to develop targeted MG therapy. Nonetheless, extensive basic science and clinical studies are required to evaluate the therapeutic potential of gene therapy.

## 4. Regenerative medicine-based approach

### 4.1 The Stem cells

Owing to ease of harvest and differentiation potential, mesenchymal stem cells (MSCs) are being evaluated for the treatment of various disorders. Previous preclinical and clinical studies have reported the therapeutic potential of MSCs in the treatment of autoimmune diseases such as multiple sclerosis (MS), systemic lupus erythematosus (SLE), rheumatoid arthritis (RA), and Crohn’s diseases (CD) [234, 235]. This is mainly attributed to the immunomodulatory properties of stem cells to address the therapeutic needs of autoimmune disease [236]. In addition, MSCs secrete extracellular vesicles (MSC-EVs), which have been shown to exhibit an immunomodulatory activity through downregulating the cytotoxic cell 1 (Tc1) and helper T cell 1 (Th1) cells and promoting the expansion of Treg cells [237]. It also significantly regulates pro-inflammatory molecules such as Tumor necrosis factor-alpha (TNF-α) and interferon-gamma (IFN-γ), along with elevating the level of anti-inflammatory molecules such as IL-10. The immuno-suppressive activity of MSCs is mainly associated with EVs which are rich in immunomodulatory biomolecules including mRNAs, miRNAs, cytokines, and chemokines [238]. Therefore, the regenerative potential of stem cells in the treatment of MG is being explored. In a seminal preclinical study, the preconditioning of human MSCs with peripheral blood mononuclear cells (PBMCs) resulted in a reduction in AChR Ab and improved immunosuppressive and anti-inflammatory activities [234]. Conditioned MSCs may regulate the expression of genes involved in immunosuppression, inhibition of cell proliferation, TNF signaling pathway, CD55 complement inhibitor, and biomolecules associated with Th1, Th17, and B cells. The intravenous injection of human bone marrow MSC (hBM-MSCs) has been shown to inhibit the proliferation of mononuclear cells and lymphocytes both *in vivo* and *in vitro* [239]. This immunosuppressive activity of hBM-MSCs could be associated with the presence of factors such as hepatocyte growth factor (HGF), transforming growth factor-beta (TGF-β), indoleamine 2,3-dioxygenase (IDO), and interleukin-10 (IL-10), IFN-γ TNF-α, IL- α or IL-1-β and its potential to inhibit mononuclear cells through cell-cell interaction and nitric oxide synthesis [240, 241]. In an EAMG rat model, the bone marrow MSCs(BMSCs) can regulate immune response by inhibiting the proliferation of T and B cells *in vitro* through secreting IDO and increasing the level of Th2, and Treg cells, and reducing the level of Th17 and Th1 cells [242]. In seven severe cases of MG, the hematopoietic stem cell transplant (HSCT) resulted in long-term symptoms and treatment-free remission in severe MG patients [243]. Similarly, a case study demonstrated that severe refractory MG has significantly recovered with HSCT without any need for medication; however, diplopia was the only remaining symptom [244]. HSCT has been shown to provide complete remission for 65 months in a severe adult female MG patient [245]. Though HSCT has been prospective various studies, still rare risk of development of autoimmune disorder post HSCT and bone marrow transplantation needs to be assessed [246, 247]. Further, it has also been observed that the effect of MSCs on T-cell proliferation was weak among MG patients [248]. MSCs also did not have any significant effect on IFN-γ production. The current studies showed that BMSCs and HSCT have the potential to provide long treatment for refractory myasthenia gravis and the conditioning of stem cells has further potential to improvise the efficacy of stem cell treatment. Notably, more studies are required to explore the application of other stem cells such as adipose-derived stem cells (ADSCs), embryonic stem cells (ESCs), and induced pluripotent stem cells (iPSCs) need to be explored critically to expand the source and choice of stem cells.

### 4.2 Exosomes: Cell-free therapeutic approach

Exosomes are extracellular vesicles (EVs) and carry many functional biomolecules such as miRNA, RNAs, proteins, etc. [249, 250]. These secreted molecules along with exosomes play an active role in regulating biological/molecular responses. In addition to biocompatibility, lower antigenicity, and immuno-genicity, the exosomes also regulate signaling pathways. This potential of exosomes makes them an attractive agent for regenerative therapies against various diseases including autoimmune disorders. Exosomes from mouse bone marrow-derived immature dendritic cells controlled the progression of MG by lowering the level of active lymphocytes for AChR, AChR antibody, and pro-inflammation cycles in the EAMG model [251]. Similarly, the statin-conditioned bone marrow dendritic cells secreted exosomes upregulated the expression of Foxp3 and Aire resulting in increased natural Treg cells leading to the potential of immunosuppression in EAMG [252]. In addition, these exosomes mediated its immunomodulation activity in organs of immune systems such as the spleen, thymus, and lymph nodes by up-regulating levels of indoleamine 2,3-dioxygenase (IDO) and Treg [253]. Further, the regulation of the FasL/Fas signaling pathway and decrease in expression of MG-related IgG antibodies in the EAMG model. Reportedly, the immature exosomes from dendritic cells overexpressing microRNA-146a regulated T cell profiles both in serum and spleen resulting in immunosuppression [254]. These pre-clinical studies indicate the immunosuppression potential of exosomes to provide a cell-free alternative therapeutic candidate for MG. However, additional studies are required to establish therapeutic procedures, clinical efficacy, and safety.

## 5. MG and COVID-19

MG patients being on immunosuppressive drugs have a high risk of COVID-19 infection [255], and the management of both COVID-19 and MG is a challenging task as clinical patterns and therapeutic outcomes vary among patients [256]. Restivo et al. also reported the development of MG symptoms to post 5-7 weeks of COVID-19 infection [257]. Similarly, a case report also suggests the development of ocular MG post-COVID-19 infection [258]. Of note, Tagliaferri et al. have reported an MG crisis post-COVID vaccination; nonetheless, the advantages of COVID vaccinations overcome the related adverse events [259]. Therefore, an additional dose of vaccine should be administered to induce an immune response [260], and a prolonged observation should be kept for MG patients post-vaccination to avoid any serious adverse events. Recent studies also indicate that both COVID-19 and MG are contributing to the increased prevalence of each other. However, immunosuppressive therapy has been considered safe and continued even after the COVID-19 infection [261, 262]. The use of Azithromycin has also been found safe [263]. Taken together, COVID -19 has the potential to induce an MG crisis, but specific care could be provided without discontinuing immunosuppressive therapy for MG patients.

## 6. Conclusion

The MG progression severely impacts the quality of life and increases the burden on the healthcare system as it demands both personal care and a dedicated therapeutic approach. Though immunosuppressant drugs and steroids are currently available treatment alternatives, the development of refractory MG and related adverse events implies a pressing need to provide a more effective and safer alternative. For the management of these pathologies, lifestyle changes, TCM/TCH, cholinergic drugs, and surgery are the traditional treatment modalities. Although, recent research has offered effective therapeutic alternatives for MG such as pyridostigmine, thymectomy, gene therapy, and TPE. Regenerative approaches like stem cells and exosomes are considered to be futuristic therapies. Nonetheless, more clinical and scientific studies are required to utilize these therapies for MG patients.
